# Chromosome groups 5, 6 and 7 harbor major quantitative trait loci controlling root traits in bread wheat (*Triticum aestivum* L.)

**DOI:** 10.3389/fpls.2023.1092992

**Published:** 2023-03-20

**Authors:** Tanushree Halder, Hui Liu, Yinglong Chen, Guijun Yan, Kadambot H. M. Siddique

**Affiliations:** ^1^ UWA School of Agriculture and Environment, The University of Western Australia, Crawley, WA, Australia; ^2^ The UWA Institute of Agriculture, The University of Western Australia, Crawley, WA, Australia; ^3^ Department of Genetics and Plant Breeding, Faculty of Agriculture, Sher-e-Bangla Agricultural University, Dhaka, Bangladesh

**Keywords:** QTL, markers, candidate genes, phenotyping, recombinant inbred lines, genomic region

## Abstract

Identifying genomic regions for root traits in bread wheat can help breeders develop climate-resilient and high-yielding wheat varieties with desirable root traits. This study used the recombinant inbred line (RIL) population of Synthetic W7984 × Opata M85 to identify quantitative trait loci (QTL) for different root traits such as rooting depth (RD), root dry mass (RM), total root length (RL), root diameter (Rdia) and root surface areas (RSA1 for coarse roots and RSA2 for fine roots) under controlled conditions in a semi-hydroponic system. We detected 14 QTL for eight root traits on nine wheat chromosomes; we discovered three QTL each for RD and RSA1, two QTL each for RM and RSA2, and one QTL each for RL, Rdia, specific root length and nodal root number per plant. The detected QTL were concentrated on chromosome groups 5, 6 and 7. The QTL for shallow RD (*Q.rd.uwa.7BL*: *Xbarc50*) and high RM (*Q.rm.uwa.6AS*: *Xgwm334*) were validated in two independent F_2_ populations of Synthetic W7984 × Chara and Opata M85 × Cascade, respectively. Genotypes containing negative alleles for *Q.rd.uwa.7BL* had 52% shallower RD than other Synthetic W7984 × Chara population lines. Genotypes with the positive alleles for *Q.rm.uwa.6AS* had 31.58% higher RM than other Opata M85 × Cascade population lines. Further, we identified 21 putative candidate genes for RD (*Q.rd.uwa.7BL*) and 13 for RM (*Q.rm.uwa.6AS*); *TraesCS6A01G020400*, *TraesCS6A01G024400* and *TraesCS6A01G021000* identified from *Q.rm.uwa.6AS*, and *TraesCS7B01G404000*, *TraesCS7B01G254900* and *TraesCS7B01G446200* identified from *Q.rd.uwa.7BL* encoded important proteins for root traits. We found germin-like protein encoding genes in both *Q.rd.uwa.7BL* and *Q.rm.uwa.6AS* regions. These genes may play an important role in RM and RD improvement. The identified QTL, especially the validated QTL and putative candidate genes are valuable genetic resources for future root trait improvement in wheat.

## Introduction

Wheat is the most important cereal crop exported in developing countries as a primary source of protein (20%) and calories (21%) (Singh, 2019; Vishwakarma et al., 2022). Climate change is stimulating multiple abiotic stresses affecting crop nutrient acquisition, grain yield and quality (Asif et al., 2019; Kumar et al., 2022), and thus threatening global crop productivity (Calleja-Cabrera et al., 2020). However, crop production needs to be at least double by 2050 to feed the future world population (Woo et al., 2021) including the current wheat production (775.6 million tons) (Alexandratos and Bruinsma, 2012; Singh, 2019; Halder et al., 2022).

Roots are pivotal for supplying water and nutrient to crops (Lynch, 2007) and for anchorage (Fitter, 2002), and thus directly affect grain yield (Lynch, 2007). However, due to the heterogeneous nature of soil environments—variations in soil texture, pH, water and nutrient (Li et al., 2021), root traits change according to environmental variations to capture edaphic resources (Grossman and Rice, 2012; Rogers and Benfey, 2015). For example, under well-watered and low moisture conditions, the shallow root system of durum wheat contributed more to yield than the deep root system while under water-limited conditions, deeper roots contributed to higher grain yields (El Hassouni et al., 2018). Furthermore, root traits vary genetically (Scheiner, 1993) and are highly heritable (Fitz Gerald et al., 2006). Therefore, genetic research on different root traits is essential for improving climate resilience and yield potential in crops (Zheng et al., 2019). Quantitative trait loci (QTL) identification is a popular approach for investigating genetic variation in quantitative traits (i.e., root traits) in many cereal crops including wheat. QTL identification requires molecular linkage maps coupled with precise phenotyping (Collard et al., 2006; Shukla et al., 2014; Soriano and Alvaro, 2019). However, obtaining reliable root data for identifying root trait associated QTL from a large number of genotypes grown in soil is challenging due to invasive nature of soil, labor intensity and is time-consuming approach (Atkinson et al., 2015; Ren et al., 2017; Chen et al., 2018). To overcome this limitation, hydroponic (Horn et al., 2016; Ren et al., 2017) and semi-hydroponic (Halder et al., 2021) systems have been used to study wheat root system, and are equally useful for QTL studies (Ren et al., 2017).

QTL studies have revealed the contribution of root trait QTL to grain yield, stress tolerance, and nutrient uptake at different growth stages in wheat (Cai et al., 2008; Bennett et al., 2012; Bai et al., 2013; Ayalew et al., 2017; Ren et al., 2017; Khalid et al., 2018; Alahmad et al., 2019; Salarpour et al., 2020), indicating the value of QTL identification in marker-assisted breeding (MAS) for root traits. For example, rooting depth (RD) and grain yield spike^–1^ were co-localized with the flanking marker *D_GA8KES401CIKOJ*–*160-BS00067285_51* on chromosome 7D (Salarpour et al., 2020). Root trait QTL of wheat seedlings correlated with QTL at maturity (Bai et al., 2013; Atkinson et al., 2015). In a doubled haploid (DH) of Rialto × Savannah, a grain yield QTL was co-located with different root traits including RD and total root length (RL) of wheat seedling on chromosome 7D (Atkinson et al., 2015). Co-localized QTL for thousand grain weight and root traits, including RL, root surface area (RSA), and root dry mass (RM) were found on chromosomes 4D, 5A and 6A (Bai et al., 2013). Therefore, genetic studies of root traits at the seedling stage might play important role in wheat yield improvement. Under normal and drought conditions, Ayalew et al. (2017) reported a stable QTL for RM on chromosome 5AL of recombinant inbred lines (RILs) of Synthetic W7984 × Opata M85. Under 35°C heat stress, a QTL for RD was found on chromosome 4D in the same population (Lu et al., 2022). Ren et al. (2017) found three significant QTL for RM on chromosomes 2A, 2D and 3B under controlled conditions and two QTL each on chromosome 4B under both low N and phosphorus (P) conditions; they also reported two QTL for RD each on chromosome 2B under both P- and N- limited conditions. A QTL for RM, *qRNAX.7A.3*, showed salt stress tolerance in the F_2_ of WTSD91 × WN-64 (Hussain et al., 2017). As wheat is a polyploid with a large genome, many QTL for root traits in wheat remain unexplored. Additionally, only a few QTL for root traits have been validated including RD (*Qrls.uwa.1AS* and *Qrls.uwa.3AL*) on chromosome 3A (Ayalew et al., 2017), RL (*QTrl.saw-2D.2*) on chromosome 2D (Zheng et al., 2019), RM on chromosome 6B (*AX-109558906*–*AX-110028322*) and chromosome 7B (*AX-95025477*–*AX-95121748*) (Meng-jiao et al., 2020).

The availability of the wheat reference genome has improved the identification of traits controlling candidate genes in QTL regions of specific chromosome and the preciseness and usefulness of QTL mapping for MAS breeding (Appels et al., 2018). In the last decade, several candidate/putative candidate genes of wheat root traits have been reported in the identified QTL regions (Wu et al., 2017; Soriano and Alvaro, 2019; Zheng et al., 2019; Li et al., 2020; Yang et al., 2021b; Griffiths et al., 2022; Li et al., 2022). For example, Wu et al. (2017) identified five putative candidate genes from QTL for root diameter (Rdia) on chromosomes 1BL, 2BL, 3BL, 3DL and 7DS under P stress conditions. *TraesCS2D02G594400* and *TraesCS2D02G594700* candidate genes were reported for RL QTL on chromosome 2D under controlled conditions (Zheng et al., 2019). However, none of the genes were functionally validated.

The genetics of wheat root traits are complex (Griffiths et al., 2022) due to the large genome (17 Gb) and polyploidy nature of bread wheat (Borrill et al., 2019). Therefore, genetic studies that identify QTL and associated genes of multiple root traits will help to understand the molecular mechanism of wheat root systems (Zheng et al., 2019), ultimately helping to develop climate-resilient, high-yielding wheat genotypes. Therefore, this research aimed to identify QTL for different root traits from RILs developed from Synthetic W79804 and Opata M85, validate key QTL in two populations with different genetic backgrounds and identify candidate genes within the flanking markers of the validated QTL.

## Materials and methods

### Plant materials

A population of 103 RILs developed from a cross between highly polymorphic parents Synthetic W7984 (*T. turgidum* cv. Altar 84/*Aegilops tauschii* Coss. line WPI 219) and Mexican spring wheat (Opata M85) accessed through the International Triticeae Mapping Initiative (Salem et al., 2007; Sorrells et al., 2011) was used for the genetic mapping study. In addition, F_2_ populations of Synthetic W7984 × Chara and Opata M85 × Cascade with different genetic backgrounds to RILs were developed to validate the phenotypic effect of two identified QTL for RD and RM, respectively.

### Experimental design and evaluation of root traits in RILs

Synthetic W7984, Opata M85, and the 103 RILs were grown in a semi-hydroponic system (Chen et al., 2011b) for 42 days in a randomized block design with four replicates for each genotype. The experimental conditions and trait measurements were the same as described by Halder et al. (2021). All plants were assessed at tiller onset [Zadoks 2.4; (Zadoks et al., 1974)], i.e. 42 days after transplanting. Briefly, the experiment was conducted in a temperature controlled (10–24°C) glasshouse at The University of Western Australia (UWA), Perth, from mid-June to late-August 2019. Wheat seedlings (4–5 cm long roots) grown in washed river sand were transplanted into bins for a semi-hydroponic system containing 35 L nutrient solution.

At harvest, the maximum depth of a plant root (RD) was measured with a ruler from its crown, and the number of nodal roots per plant (NNR) was counted manually. After capturing photographs of the root system using a portable photographing system, the root system were separated from the shoot. Root sections (≤ 20 cm) were scanned at 400 dpi using a desktop scanner (Epson Perfection V800/850) to determine other root traits—RL (sum of all root length types), Rdia, RSA and root diameter length (RDCL) of fine roots (root diameter< 0.25 mm) and coarse roots (root diameter > 0.25 mm)—were measured using WinRHIZO Pro software (v2009, Regent Instruments Inc., Montreal, QC, Canada). Specific root length (SRL) was calculated as the RL per unit of RM, and root length intensity (RLI) was the RL per unit of RD. Root growth rate is the RD per day. RM is the weight of the whole root system after air-forced oven drying (65°C for 72 h). Further, using the phenotypic data, broad-sense heritability (H^2^) of the root traits was calculated as:

H^2^ = $\sigma_{G}^{2}/\left[\sigma_{G}^{2}+\;\frac{\sigma_{E}^{2}}{n}\right]$ , where $\sigma_{G}^{2}\;$ is genotypic variance (mean sum of squares of a trait) and $\sigma_{E}^{2}$ is environmental variance (residual mean sum of square) from the analysis of variance, and *n* is replication number per genotype (4) (Wu et al., 2013; Ben Sadok et al., 2015).

### QTL mapping

Molecular marker data and the linkage map of the Synthetic W7984 × Opata 85 RIL mapping population were accessed from the GrainGenes database (https://wheat.pw.usda.gov/cgi-bin/GG3/report.cgi?class=mapdata&name=Wheat%2C%20Synthetic%20x%20Opata%2C%20BARC). The linkage map comprised 1,476 simple-sequence repeats (SSR) and restriction fragment length polymorphism (RFLP) markers distributed across 21 linkage groups. Among the available markers, 1,018 with known chromosomal locations were used for QTL mapping of the target root traits. The genetic map spanned a length of about 500 cM with an average marker density of 1 cM after filtering the 20% missing values from the dataset.

The composite interval mapping method in Windows QTL Cartographer V2.5_011 was used to identify root traits QTL; the logarithm of odds (LOD) threshold value was set to ≥ 2.5 based on 500 and 1,000 permutations at the 5% significance level. LOD > 2.5 indicate the presence of significant QTL in a particular genomic region for an individual trait. The square of the partial correlation coefficient (R^2^) estimates the phenotypic variance of a single QTL (Balakrishnan et al., 2020). The sequences of the SSR and RFLP flanking markers (left-and right-hand sides closest to the QTL regions) were identified from GrainGenes database (https://wheat.pw.usda.gov/cgi-bin/GG3/browse.cgi?class=marker; accessed on 05 October 2022) and/or NCBI database (https://www.ncbi.nlm.nih.gov/; 05 October 2022), respectively. Further the sequences were blasted in JBrowse (https://urgi.versailles.inra.fr/blast/?dbgroup=wheat_iwgsc_refseq_v1_chromosomes&program=blastn) with the wheat reference genome RefSeq v1.0 to identify the physical position of the markers. The graphical representation of the QTL was drawn using MapChart 2.32 software.

### Marker validation using validation populations

One-third of an individual seed (excluding the embryo) was used to extract the genomic DNA of Synthetic W7984, Opata M85, Chara, Cascade, and the F_2_ of Synthetic W7984 × Chara and Opata M85 × Cascade. The remaining seed with embryo was preserved in the cold room for seed germination to validate the phenotypic effect of the targeted QTL. The one-third seed part was crushed manually using a small hammer, and then crushed further with a SPEX^®^ SamplePrep 2010 GenoGrinder at 1,400 rpm for 2 minutes for DNA extractions following the cetyl trimethyl ammonium bromide (CTAB) method. The extracted DNA was suspended in 0.1× TE buffer (pH 8.0) for storage. DNA concentrations were measured by NanoDrop (NanoDrop-1000 spectrophotometer) and Qubit 2.0 fluorometer using the Qubit dsDNA BR (Broad-Range) Assay Kit. The primers (forward and reverse) for SSR marker *Xbarc50* were obtained from Sigma-Aldrich (Sigma-Aldrich Pty Ltd., NSW, Australia). DNA primers of *Xgwm334* (forward (5´) dyed with fluorescent PET) were obtained from Alpha ADN (225 Bridge CP 4023, Montreal, Quebec H3C 0J7, Canada: http://www.alphaadn.com/contact.html).

An EmeraldAmp^®^MAX HS PCR Master Mix reaction mixture (15 µL) containing 20 ng template DNA of Synthetic W7984 and Synthetic W7984 × Chara populations, 0.2 µM of each forward and reverse primers was amplified in a thermocycler (Eppendorf Mastercycler EP Gradient S) to validate the *Q.rd.uwa.7BL* with a flanking marker *Xbarc50*. The annealing temperature of the marker (53°C) was found in GrainGenes (https://wheat.pw.usda.gov/cgi-bin/GG3/report.cgi?class=marker&name=&id=86860). The PCR conditions were 98°C for 1 min, 35 cycles of denaturation at 98°C for 10 sec, annealing at 53°C for 30 sec, elongation at 72°C for 1 min kb^–1^ and final extension (Taq polymerase) at 72°C for 5 min. The PCR products were run on a 2.5% agarose gel electrophoresis using GelRed™ (1:10 ratio) at 120 V for 1 h 20 min. The experiment was conducted at the genetics and molecular genetics laboratories at the UWA School of Agriculture and Environment.

A DNA fragment analysis was undertaken using the Applied Biosystems Genetic Analyzer at Biodiversity Conservation Centre, Kings Park, WA, to validate *Q.rm.uwa.6AS* with a flanking marker *Xgwm334*. The annealing temperature of the marker was determined by a gradient PCR using RT-PCR. The master mix (1rxn) for gradient PCR was 5 µL SYBR Green, 1.5 µL of each forward and reverse primer and 2 µL template DNA of Opata M85 and Cascade. Using PCR conditions at 98°C for 2 min, 40 cycles denaturation at 98°C for 10 sec, a range of annealing at 52–67°C for 45 sec, elongation at 72°C for 30 sec kb^–1^ and final extension at 72°C for 5 min, the best annealing temperature for the marker was set at 58°C. Singleplex PCR of template DNAs (20 ng) from the Opata M85 × Cascade populations and both parents was done in the wheat genetics laboratory at UWA. The master mix (1×) for a singleplex PCR was 3.52 µL PCR grade water, 2 µL 5× buffer, 0.8 µL MgCl_2_ (25mM), 0.08 µL Taq polymerase (0.04 u µL^-1^), 0.8 µL of each fluorescent forward primer, and reverse primer, and 2 µL of template DNA (≥ 2 ng µL^-1^). Singleplex PCR conditions were 94°C for 5 min, 40 cycles denaturation at 94°C for 30 sec, annealing at 58°C for 1min, elongation at 72°C for 45 sec kb^–1^, final extension at 72°C for 7 min and hold at 10°C. A multiplex PCR was done using 1 µL PCR product mixed with 9 µL highly deionized (Hi-Di) formamide with LIZ Size Standard for fragment analysis in an ABI sequencer.

Further, 1 µL PCR product was mixed with 9 µL Hi-Di with LIZ Size Standard for fragment analysis using capillary electrophoresis in an Applied Biosystems 3500 series Genetic Analyzer at Biodiversity Conservation Centre, Kings Park, WA. The DNA fragment size were identified by analyzing the electrogram from SeqPartitioner, Geneious plugin.

Homozygous (AA from Synthetic W7984 or Opata M85, and BB from Cascade or Chara alleles) individuals were identified by comparing differences in band size in the agarose gel and the DNA fragment size of their respective parents. Selected individuals were grown in a semi-hydroponic system in a controlled environment (day/night 24°C, 14°C) as described above. The average RD and RM of the genotypes of two allelic combinations (AA and BB) were compared using a student’s t-test at 0.05% significance level.

### Statistical analysis

Phenotypic data were analyzed using GenStat statistical software 19th edition, with the frequency analysis done in SPSS Version 28.0.0 (142) (https://www.ibm.com/support/pages/node/6525830).

### Potential candidate gene identification

Potential high confidence candidate genes for root traits were identified in the two QTL considered for validation. The physical position of the flanking markers of the QTL was found in the GrainGenes wheat database (https://wheat.pw.usda.gov/cgi-bin/GG3/browse.cgi?class=marker), and blasted in the JBrowse (http://www.wheatgenome.org/Tools-and-Resources/Sequences, accessed on 01 June 2022) wheat genome browser with RefSeq v1.0 to identify the candidate genes on the QTL region.

Further, the gene functions were identified in the International Wheat Genome Sequencing Consortium (IWGSC) RefSeq v1.0 website (https://wheaturgi.versailles.inra.fr/Seq-Repository/Annotation, accessed on 20 June 2022) (Appels et al., 2018). Genes involved in root growth and development from other studies were considered putative candidate genes. The biological functions of the individual genes were obtained from Uniprot (https://www.uniprot.org/?-id+2fYRW1ChXSa+-fun+Pagelibinfo+-info+TREMBL). *WheatExp* revealed expression of the candidate genes on root tissues in other wheat cultivars (Pearce et al., 2015) (http://www.wheat-expression.com/; accessed on 01 December 2022). Gene expression levels for the candidate genes in different wheat tissues, including roots were downloaded from *WheatExp*. Further, gene expression in root tissue was filtered, with the highest expression level considered for this study.

## Results

### Phenotypic evaluation

**Figure 1:**
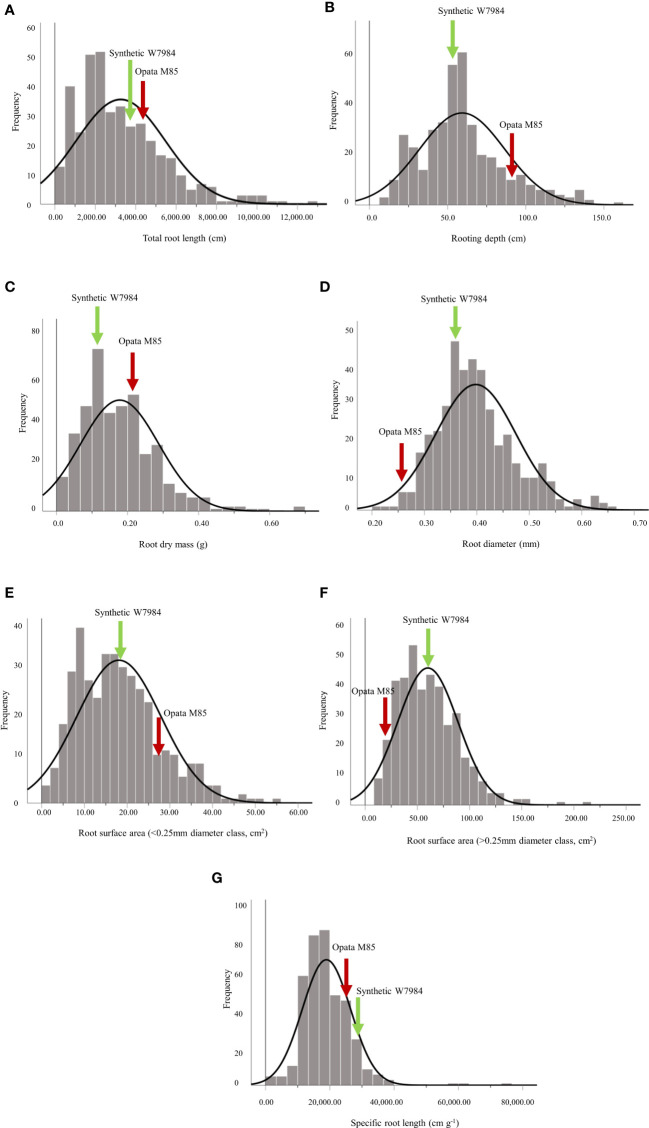
Distribution of **(A)** total root length (cm), **(B)** rooting depth (cm), **(C)** root dry mass (g), **(D)** root diameter (mm), **(E)** root surface area of fine roots (root diameter< 0.25 mm, cm^2^), **(F)** root surface area of coarse roots (root diameter > 0.25 mm, cm^2^), and **(G)** specific root length (cm g^-1^) of 103 recombinant inbred lines and their parents, Synthetic W7984 and Opata 85. The green and red arrows indicate the phenotypic performance of Synthetic W7984 and Opata M85, respectively.

Root traits of the Synthetic W7984 × Opata M85 RILs and their parents varied considerably (**Table 1**). Opata M85 had higher RL, RD, RM, root surface area of fine roots (root diameter < 0.25 mm, cm^2^; RSA2), and total length of coarse roots (root diameter < 0.25 mm, cm; RDCL2) than Synthetic W7984, while Synthetic W7984 had higher root surface area of fine roots (root diameter > 0.25 mm, cm^2^; RSA1), SRL, and total length of coarse roots (root diameter > 0.25 mm, cm; RDCL1) than Opata M85 (**Table 1**). For the RILs, RL, RD, RM, Rdia, RSA1, RSA2, and SRL ranged from 173.10–12,783 cm, 8.00–158 cm, 0.03–0.44 g, 0.21–0.66 mm, 10.07–216.20 cm^2^, 1.16–55.17 cm^2^, and 2,150–74,013 cm g^–1^, respectively (**Table 1**). Transgressive segregation with approximately normal distribution for various root traits (RL, RD, RM, Rdia, RSA1, RSA2, and SRL) between the RILs and the parents was detected (**Figure 1**). H^2^ was high (84.5–92.1%) for all root traits except RLI (data not shown).

**Table 1:** Variations in root traits of recombinant inbred lines (RILs) and their parents, Synthetic W7984 and Opata M85.

Traits	Parents (mean ± SE)		RILs			Skewness	Kurtosis
	Synthetic W7984	Opata M85	Mean ± SE	Minimum	Maximum		
RL (cm)	3551.00 ± 517.40	5331.00 ± 382.30	3232.00 ± 111.14	173.10	12783.00	1.22	1.67
RD (cm)	51.70 ± 0.51	83.37 ± 1.2	59.44 ± 1.39	8.00	158.00	0.85	0.81
RM (g)	0.13 ± 0.03	0.22 ± 0.05	0.18 ± 0.00	0.03	0.44	1.41	3.43
Rdia (mm)	0.35 ± 0.03	0.27 ± 0.02	0.40 ± 0.00	0.21	0.66	0.78	0.78
RSA1 (cm^2^)	68.93 ± 8.56	33.38 ± 10.03	60.17 ± 1.45	10.07	216.20	1.89	2.57
RSA2 (cm^2^)	18.72 ± 2.69	26.29 ± 1.59	18.03 ± 0.49	1.16	55.17	0.79	0.47
SRL (cm g^-1^)	28817.00 ± 4267.00	26219.00 ± 3898	18816.35 ± 373.70	2150.00	74013.00	2.00	11.13
RSR	0.40 ± 0.07	0.41 ± 0.02	0.381 ± 0.01	0.02	2.44	4.88	39.69
RDCL1 (cm)	520.10 ± 66.01	261.80 ± 78.93	421.30 ± 9.97	62.49	1202.00	0.77	0.27
RDCL2 (cm)	452.3 ± 80.53	632.0 ± 73.43	417.90 ± 11.27	25.92	1213.00	0.87	1.22
NNR	3.00 ± 0.58	3.00 ± 0.33	4.00 ± 0.09	0.00	11.00	0.68	0.80
RGR (cm day^-1^)	1.23 ± 0.01	1.99 ± 0.03	1.41 ± 0.03	0.19	3.76	0.85	0.81
RLI	68.63 ± 9.78	63.93 ± 4.35	54.02 ± 1.31	3.83	179.30	0.96	1.32

RL, total root length; RD, rooting depth; RM, root dry mass; Rdia, root diameter; RSA1, root surface area of coarse roots (root diameter > 0.25 mm); RSA2, root surface area of fine roots (root diameter < 0.25 mm); SRL, specific root length; RSR, root-shoot ratio; RDCL1, total length of coarse roots (root diameter > 0.25 mm); RDCL2, total length of fine roots (root diameter < 0.25 mm); NNR, number of nodal roots per plant; RGR, root growth rate; RLI, root length intensity. SE, standard error for mean.

### QTL mapping

The permutation tests identified 14 and nine QTL for eight root traits, with LOD scores ≥ 2.5 in CIM at 500 and 1,000 permutations, respectively. However, considering that root system architecture is complex and governed by many genes of small effect (Sharma et al., 2011), the study considered the QTL identified at 500 permutations. Most of the QTL were distributed on chromosome groups 5, 6 and 7, except 5B and 7D chromosomes. The QTL for RSA1 were found on chromosome 2A and 3B (**Table 2**). Opata M85 contributed alleles to all the QTL for RL, RM, RSA1 and NNR and a QTL for RD (*Q.rd.uwa.5DL*), and Synthetic W7984 contributed all other alleles.

**Table 2:** QTL for eight root traits in recombinant inbred lines (RILs) of Synthetic W7984 × Opata M85 identified by composite interval mapping (CIM) at the logarithm of odds (LOD) threshold ≥ 2.5.

Traits	QTL name	QTL position (cM)	LOD	R^2^ (%)	Additive	Flanking markers	Physical position of markers (Mb)
RL	*Q.rl.uwa.5AL*	34.20	2.65	8.88	–481.93	*Xbarc1-5A–Xbcd157-5A*	326.06–444.94
RD	*Q.rd.uwa.5AL*	72.30	3.13	11.03	6.62	*Xbarc151-5A–Xbarc230-5A*	315.92–558.34
	*Q.rd.uwa.5DL*	120.50	4.14	16.52	–8.27	*Xbarc93-5D– Xbarc322-5D*	473.35–497.83
	*Q.rd.uwa.7BL*	123.40	4.00	13.14	7.16	*Xgwm611-7B–Xbarc20-7B*	329.79–700.63
RM	*Q.rm.uwa.6AS*	6.80	2.80	9.49	–0.02	*Xbcd21-6A–Xcmwg652-6A*	8.00–22.02
	*Q.rm.uwa.7AL*	124.60	2.87	12.86	–0.03	*Xcdo347-7A–Xbarc275-7A*	2.00–603.08
Rdia	*Q.rdia.uwa.6AL*	74.40	2.65	9.02	0.01	*Xbarc107-6A–Xmwg934-6A*	495.11–583.27
RSA1	*Q.rsa1.2AS*	49.60	3.08	9.89	–2.17	*Xcdo57-2A–Xbarc231-2A*	15.00–367.14
	*Q.rsa1.3BS.1*	8.00	3.43	11.84	–2.31	*Xbarc75-3B–Xbarc133-3B*	3.40–7.61
	*Q.rsa1.3BS.2*	18.00	2.79	11.07	–2.23	*Xbarc133-3B–Xgwm493-3B*	7.61–13.94
RSA2	*Q.rsa2.5DL*	125.50	2.99	10.48	6.17	*Xbarc93-5D–Xcdo346-5D*	139.73–473.35
	*Q.rsa2.6BS*	35.00	2.77	8.54	5.49	*Xrz995-6B–Xbcd102-6B*	3.52–646.04
SRL	*Q.srl.uwa.6DS*	44.00	4.03	13.64	0.00	*Xmwg549-6D–Xbarc196-6D*	0.00–59.74
NNR	*Q.nnr.uwa.5AL*	128.30	3.63	14.71	–0.52	*Xgwm595-5A–Xgwm410-5A*	659.13–680.07

RL, total root length (cm); RD, rooting depth (cm); RM, root dry mass (g); Rdia, root diameter (mm); RSA1, root surface area of coarse roots (root diameter > 0.25 mm, cm^2^); RSA2, root surface area of fine roots (root diameter< 0.25 mm, cm^2^); SRL, specific root length (cm g^-1^); and NNR, number of nodal roots per plant. QTL position, QTL position on the linkage map based on GrainGene (https://wheat.pw.usda.gov/GG3/node/876); LOD, logarithm of odds; R^2^, % of phenotypic variance explained by an individual QTL. An additive value indicates the parental contribution of the QTL; a negative value indicates that the trait-enhancing allele is contributed by Opata M85, and a positive value indicates that the trait-enhancing allele is from Synthetic W7984, and zero indicates that either parent could contribute to the trait; Mb, mega base pair.

**Figure 2:**
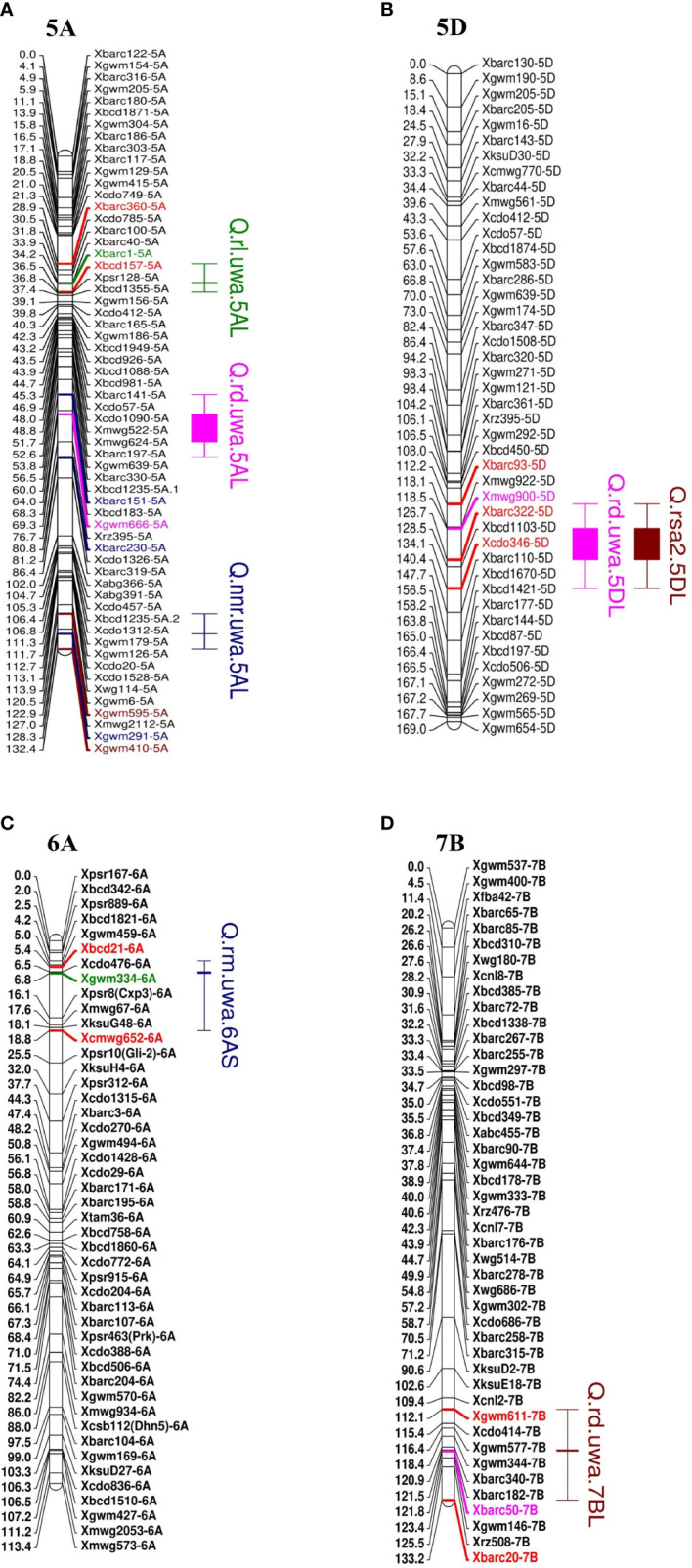
Mapping of important QTL for root traits in wheat in the Synthetic W7984 and Opata 85 RIL population **(A)** total root length (RL, cm), rooting depth (RD, cm), and nodal roots per plant (NNR) **(B)** rooting depth and root surface area of fine roots (root diameter < 0.25 mm, RSA2, cm^2^), **(C)** root dry mass (RM), and **(D)** rooting depth of recombinant inbred lines of Synthetic W7984 × Opata 85. Bars and caps indicate the QTL with LOD > 2.5. Red markers are flanking markers of different colours in individual chromosomes that represent the tightly linked marker of the respective QTL.

Seven of the QTL on chromosome groups 5, 6 and 7 explained more than 10% phenotypic variance (R^2^). Three QTL for RD (R^2^ = 11.03–16.52%; LOD 3.14–4.14) were identified and distributed on the long arms of chromosomes 5A, 5D and 7B (**Figure 2**). Synthetic W7984 contributed alleles to *Q.rd.uwa.5AL* and *Q.rd.uwa.7BL*, and Opata M85 contributed alleles to *Q.rd.uwa.5DL*. Among the 11 QTL, *Q.rd.uwa.5DL* had the highest LOD (4.14) and R^2^ (16.52) values. For the two QTL for RM (both contributed by Opata M85), one was distributed on the long arm of chromosome 7A (R^2^ = 12.86%; LOD = 2.87) and the other on the short arm of chromosome 6A (R^2^ = 9.49%; LOD = 2.80) (**Figure 2C**); the LOD and R^2^ were same for both 500 and 1,000 permutations. QTL for RL and NNR occurred on the long arm of chromosome 5A. Two QTL for RSA2 were detected on chromosomes 5DL (**Figure 2B**) and 6BS, and QTL for SRL was identified on chromosome 6DS.

Co-location of *Q.rd.uwa.5DL* and *Q.rsa2.5DL* were identified at 120.50 cM (**Figure 2B**). The flanking marker interval was 112.2–126.7 cM for *Q.rd.uwa.5DL* and 112.2–134.1 cM for *Q.rsa2.5DL*.

### QTL validation

*Xbarc50*, the closely linked marker of *Q.rd.uwa.7BL*, showed polymorphism between Synthetic W7984 and Chara in agarose gel electrophoresis. *Xgwm334*, the closely linked marker of *Q.rm.uwa.6AS*, showed polymorphism between Opata M85 and Cascade in DNA fragment analysis. The fragment size of the parents (**Table 3**) was used to score the randomly selected F_2_ populations of the two validation population lines—19 lines for Synthetic W7984 × Chara, and 13 lines for Opata M85 × Cascade. The Synthetic W7984 × Chara hybrids (F_2_) were divided into two groups (only homozygous lines). The group containing the negative allele from Synthetic W7984 (*Q.rd.uwa.7BL*) had a significantly (P < 0.01) shorter (52%) RD than Chara (**Table 4**). Similarly, in the Opata M85 × Cascade hybrids, the group containing the positive allele from Opata M85 (*Q.rm.uwa.6AS*) had a significantly (P < 0.01) higher (31.58%) RM than Cascade (**Table 4**).

**Table 3:** Fragment size of two SSR markers, with polymorphism among parental lines (Synthetic W7984, Opata M85, Chara, and Cascade) of the validation population, related to QTL for rooting depth (RD) and root dry mass (RM).

Markers	Fragment size (bp)	Synthetic	Chara	Opata	Cascade	Traits considered	Related QTL
*Xbarc50-7B*		120	140	Null	Null	RD	*Q.rd.uwa.7BL*
*Xgwm334-6A*		130	140	145	150	RM	*Q.rm.uwa.6AS*

bp, base pair.

**Table 4:** Validation of quantitative trait loci (QTL) for rooting depth (RD) and root dry mass (RM) identified from Synthetic W7984 × Opata M85 recombinant inbred line (RIL) population in F_2_ populations of Synthetic W7984 × Chara and Opata M85 × Cascade.

Traits	F_2_ populations	Markers	AA	BB	P-value
RD (cm)	Synthetic W7984 × Chara	*Xbarc50-7B*	13.31 ± 7.47	27.83 ± 12.52	0.00**
RM (g)	Opata M85 × Cascade	*Xgwm334-6A*	0.19 ± 0.04	0.13 ± 0.05	0.00**

AA homozygous alleles from Synthetic W7984 or Opata M85, BB homozygous alleles from Chara or Cascade; Student’s t-test (P < 0.05) was used to identify differences between lines in the population with distinct allele peaks; **, significant at P < 0.01.

### Candidate gene identification

The 329.79–700.63 Mb mapping interval of *Q.rd.uwa.7BL* contained 2,323 genes, with the functions of 215 genes associated with the wheat root system (Halder et al., 2022) (**Supplementary Table S1**). Twenty-one genes were putative candidate genes for root traits and abiotic and biotic stress tolerance in wheat, reported earlier in other crops and *Arabidopsis* (**Table 5**). The 8.00–22.02 Mb mapping interval of *Q.rm.uwa.6AS* contained 387 genes, with the functions of 34 genes reported in the wheat root system (Halder et al., 2022) (**Supplementary Table S1**) and 13 were putative candidate genes for root traits and abiotic and biotic stress tolerance in wheat (**Table 5**). The in-silico expression study identified, high expression levels of the candidate genes in wheat cultivars ‘Chinese Spring,’ ‘Nulliterea Chinese Spring,’ ‘Azhurnaya,’ and ‘N1DT1A’ (**Supplementary Table S2**). Among the putative candidate genes from both QTL, *TraesCS7B01G374800*, had the highest expression level (log2 of transcripts per million: 360.65) in the roots of ‘Chinese Spring’. In the roots of ‘Azhurnaya,’ *TraesCS7B01G404000*, *TraesCS7B01G368400*, and *TraesCS6A01G026500* had high-expression levels.

**Table 5:** Genes located within the two quantitative trait loci (QTL) and the encoded proteins related to root traits and abiotic stress tolerance in crops and *Arabidopsis*.

QTL	Peak marker and associated gene names	Position RefSeq V1.0 (bp) Start End		Gene length (bp)	Positional distance of the gene from the peak marker (Mb)	Encoded protein	The biological process from UniProtKB Gene ID in wheat	Root traits of crops and/or plant	References
*Q.rm.uwa.6AS*	*Xgwm334-6A*	9249275	9249294						
	*TraesCS6A01G020400*	9482380	9486337	3958	0.23	Histidine-containing phosphotransfer protein	A0A3B6NJQ7: Cytokinin-activated signaling pathway; phosphorylation	Primary root growth of *Arabidopsis*; root growth of barley under phosphorus limited/resupply conditions.	(Nishimura et al., 2004; Hutchison and Kieber, 2007; Ma et al., 2021)
	*TraesCS6A01G021000*	10065877	10066564	734	0.82	Thaumatin-like protein	A0A3B6NHJ4: Not specified	Root development in barley; salt stress tolerance in *Arabidopsis*.	(Misra et al., 2016; Iqbal et al., 2020)
	*TraesCS6A01G022800*	11413976	11414807	832	2.16	Hydroxycinnamoyl-CoA quinate/shikimate transferase	A0A3B6NIZ3: Not specified	Root development in rice; mercury stress tolerance in rice.	(Chen et al., 2012; Song et al., 2020)
	*TraesCS6A01G024400*	12131874	12133343	1470	2.88	3-ketoacyl-CoA synthase	A0A3B6NJU8: Fatty acid biosynthetic process	Root growth and development, and drought and salinity stress tolerance of *Arabidopsis*; root growth and length in barley.	(Lee et al., 2009; Weidenbach et al., 2015; de Silva et al., 2021; Kim et al., 2021)
	*TraesCS6A01G026500*	13071476	13080970	9495	3.82	Lysine-specific demethylase 3B	A0A3B6NK41: Cellular macromolecule metabolic process; metabolic, biosynthesis and cellular process; protein modification process	Seminal root length and drought tolerance in maize; root elongation and salt tolerance in soybean.	(Sun et al., 2019; Guo et al., 2020)
	*TraesCS6A01G036800*	17956048	17958722	2675	8.71	Subtilisin-like protease	A0A3B6NHY8: Proteolysis	Root development and elongation in *Arabidopsis*.	(Neuteboom et al., 1999; Sénéchal et al., 2014)
	*TraesCS6A01G033500*	16448568	16449328	761	7.20	Germin-like protein	A0A3B6NK48: Not specified	Root development and biotic/abiotic stress tolerance in rice and *Arabidopsis*; salt stress tolerance in barley.	(Hurkman et al., 1991; Li et al., 2016)
	*TraesCS6A01G016000*	7986686	7989822	3137	1.26	Mitochondrial transcription termination factor-like	A0A3B6NIS6: Developmental process; transcription regulation	Root growth and rooting depth of *Arabidopsis*; salt and drought stress tolerance in *Arabidopsis*; abundant expression of *ZmTERF11*, *Zmsmk3* in maize.	(Kim et al., 2012; Zhao et al., 2014; Robles et al., 2015; Robles et al., 2018; Pan et al., 2019)
	*TraesCS6A01G016400*	8170486	8171640	1155	1.08				
	*TraesCS6A01G016500*	8174985	8176124	1140	1.07				
	*TraesCS6A01G016600*	8197311	8197880	570	1.05				
	*TraesCS6A01G016700*	8200649	8202396	1748	1.05				
	*TraesCS6A01G016800*	8208530	8212635	4106	1.04				
*Q.rd.uwa.7BL*	*Xbarc50-7B*	172356933	172356909						
	*TraesCS7B01G404000*	672385976	672389869	3894	500.03	Glutaredoxin	A0A3B6STD0: Cellular response to oxidative stress	Root growth and arsenic and salt stress tolerance in rice; primary root growth in *Arabidopsis*.	(Patterson et al., 2016; Verma et al., 2020; Verma et al., 2021)
	*TraesCS7B01G254900*	470835221	470837623	2403	298.48	3-ketoacyl-CoA synthase	A0A3B6SM99: Fatty acid biosynthetic process	Root growth and development and drought tolerance of *Arabidopsis*; root growth and length in barley.	(Lee et al., 2009; Weidenbach et al., 2015; Kim et al., 2021)
	*TraesCS7B01G317300*	567591057	567595950	4894	395.23	Shikimate kinase 1	A0A3B6SKV8: Not specified	Root growth, development, and root density in maize.	(Zanin et al., 2015)
	*TraesCS7B01G368400*	632729301	632730767	1467	460.37	[F-actin]-methionine sulfoxide oxidase MICAL2	A0A1D6SDJ6: Not specified	Rooting depth and drought tolerance in *Arabidopsis*.	(Li et al., 2012)
	*TraesCS7B01G372600*	638485451	638489190	3740	466.13	OTU domain-containing protein	A0A3B6SIU1: Protein deubiquitination	Drought tolerance in rice.	(Wang et al., 2011; Kohli et al., 2012)
	*TraesCS7B01G446200*	709510490	709514663	4174	537.15	Pathogenesis-related thaumatin family protein	A0A3B6ST54: Defense response	Root development in barley; salt stress tolerance in *Arabidopsis*.	(Misra et al., 2016; Iqbal et al., 2020)
	*TraesCS7B01G255900*	474408771	474413621	4851	302.05		A0A3B6SJ44: CAAX-box protein processing		
	*TraesCS7B01G283300*	517391900	517395207	3308	345.03	Subtilisin-like protease	A0A3B6SNC4: Proteolysis	Root development and elongation in *Arabidopsis*.	(Neuteboom et al., 1999; Sénéchal et al., 2014)
	*TraesCS7B01G391700*	658173522	658175923	2402	485.82				
	*TraesCS7B01G289600*	525025227	525027470	2244	352.67	Germin-like protein	A0A3B6SNK3: Not specified	Root development and biotic/abiotic stress tolerance in rice and *Arabidopsis*; salt stress tolerance in barley.	(Hurkman et al., 1991; Li et al., 2016)
	*TraesCS7B01G337900*	592534007	592534932	926	420.18				
	*TraesCS7B01G338300*	592718485	592719389	905	420.36				
	*TraesCS7B01G338400*	592807798	592808848	1051	420.45				
	*TraesCS7B01G338500*	592867670	592868665	996	420.51				
	*TraesCS7B01G351500*	608380045	608380790	746	436.02				
	*TraesCS7B01G351600*	608392307	608393057	751	436.04				
	*TraesCS7B01G351700*	608405328	608406075	748	436.05				
	*TraesCS7B01G351800*	608415055	608416000	946	436.06				
	*TraesCS7B01G374800*	639926003	639927050	1048	467.57				
	*TraesCS7B01G374900*	639945719	639946519	801	467.59				
	*TraesCS7B01G442000*	706850893	706853891	2999	534.49				

bp, base pair; Mb, mega base pair.

## Discussion

A semi-hydroponic phenotyping system was used for phenotyping a RIL mapping population of Synthetic W7984 × Opata M85 and to identify QTL for different root traits in wheat. A total of 14 QTL for eight root traits were detected on nine wheat chromosomes, with two important QTL validated in two independent F_2_ populations. The QTL identified were concentrated in wheat chromosome groups 5, 6 and 7. Several putative genes located in the QTL region were identified for the molecular breeding of root traits.

### Phenotypic analysis of root traits

The semi-hydroponic system used in this study offered an excellent opportunity to acquire reliable root trait data with high accuracy and repeatability (Chen et al., 2020). In our recent study, root trait variability of 184 bread wheat genotypes originating from 37 countries was characterized in the same semi-hydroponic phenotyping system (Chen et al., 2020), followed by validation of genotypes with contrasting root systems in soil-filled rhizoboxes (Figueroa-Bustos et al., 2018). The consistent ranking of genotypes for some important root traits in the semi-hydroponic system and soil conditions indicates the reliability of the phenotyping study for root studies, as confirmed in other crop species, such as narrow-leafed lupin (Chen et al., 2011a; Chen et al., 2014), barley (Wang et al., 2021), and soybean (Liu et al., 2021; Salim et al., 2022). The wheat lines used in this study will be examined further under the field conditions. Significant phenotypic variation for all measured root traits in the biparental population indicates the successful identification of the polygenic trait (**Table 1**). Further, the continuous distribution of different root traits such as RL, RD, RM, Rdia, RSA1, RSA2 and SRL (**Figure 1**) indicates that the genetic architecture of individual trait has many genes responsible for the variation. Similarly, high broad-sense heritability (> 80%) for all root traits except RLI (data not shown) indicates the potential for selecting these traits in future wheat breeding. Earlier studies demonstrated significant phenotypic variation in root traits under drought stress (Ayalew et al., 2017), heat stress (Lu et al., 2022), and waterlogging stress (Yu and Chen, 2013) of the same population suggesting that the population in the current study is suitable for genetic mapping of root traits.

Understanding root trait variation is essential for manipulating the traits according to the soil and environmental conditions to improve stress tolerance and yield in wheat. For example, a large wheat root system (in terms of RL and RM) was beneficial for higher grain yield, capturing water and nutrient from sandy soil under well-watered conditions (Palta and Watt, 2009; Palta et al., 2011). However, large root systems reduced yield at terminal drought due to lower (59%) water use efficiency than shallow root systems (Figueroa-Bustos et al., 2020). RM is another important root trait positively correlated with grain yield under drought stress (Ehdaie et al., 2012). SRL (ratio of RL and RM) is an indicator of utilization in nutrient uptake. Wheat genotypes with large SRL and more fine roots take up nutrient and water from the subsoil and contribute to high yield (Chen et al., 2020). Similarly, Rdia significantly correlates with wheat yield and P acquisition (Atta et al., 2013; Nahar et al., 2022).

### QTL for root traits in wheat

Several studies have reported the genetics of RL (Ibrahim et al., 2012; Atkinson et al., 2015; Danakumara et al., 2021; Yang et al., 2021a; Li et al., 2022), RD (Liu et al., 2013; Xu et al., 2013; Ayalew et al., 2017; Ren et al., 2017; Salarpour et al., 2020; Danakumara et al., 2021) and RM (Yu and Chen, 2013; Acuna et al., 2014; Meng-jiao et al., 2020; Danakumara et al., 2021) under different environmental conditions. In our study, we discovered 11 QTL on wheat chromosome groups 5, 6 and 7 responsible for root traits contributed by the two parents, Synthetic W7984 and Opata M85. Both parents could contribute favorable alleles for root traits (Bhoite et al., 2018) (**Table 2**).

Identification of putative QTL alone is insufficient for trait improvement using MAS. Therefore, validating QTL—testing the allelic effect in populations other than the original population—is essential to eliminate statistical error (Langridge et al., 2001). In this study, we validated two flanking markers for RD and RM QTL in populations different than the original population used for QTL identification.

**Figure 3:**
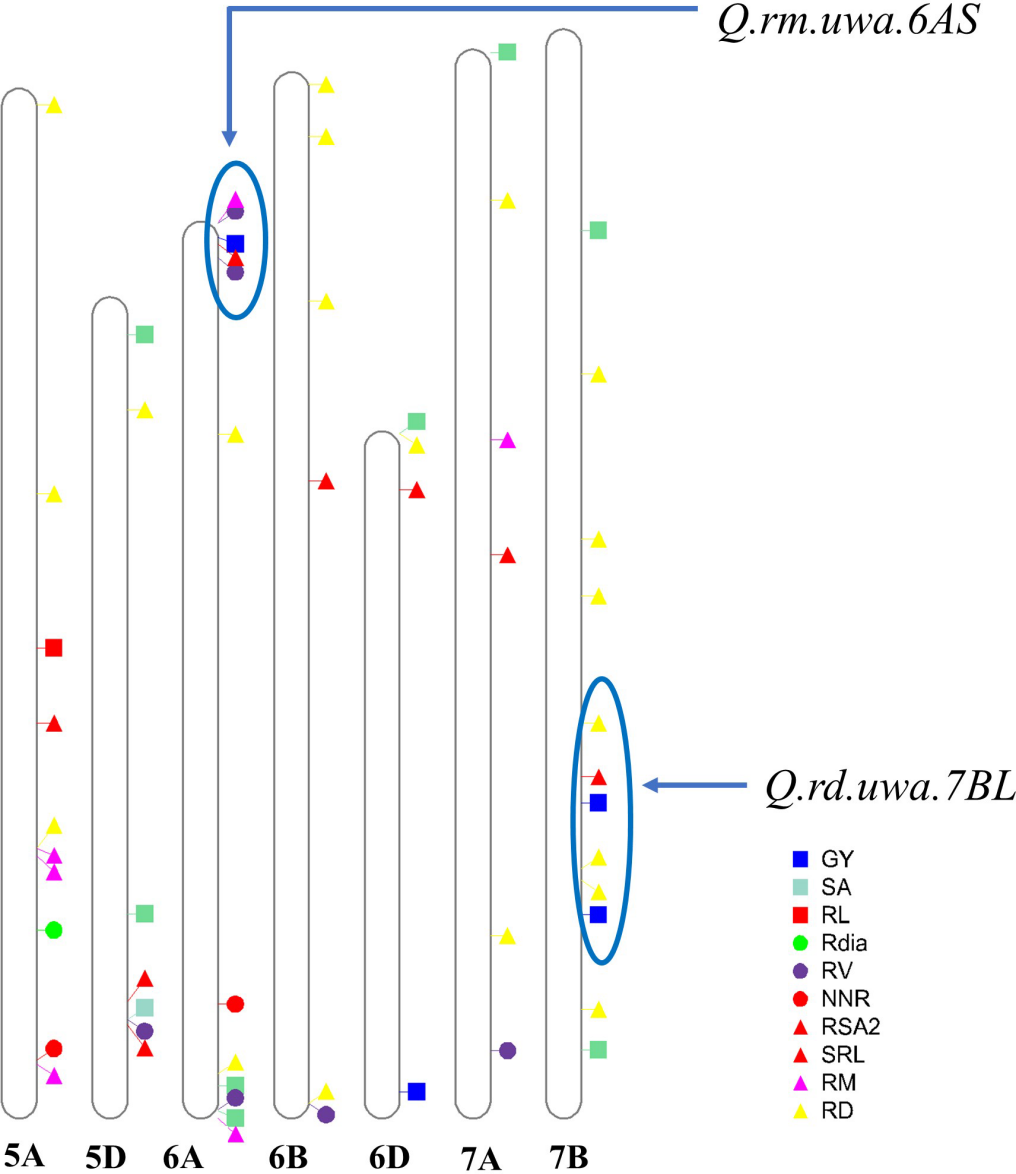
Quantitative trait loci (QTL) identified in this study and previous studies (Ibrahim et al., 2012; Ren et al., 2012; Liu et al., 2013; Xu et al., 2013; Atkinson et al., 2015; Ehdaie et al., 2016; Ayalew et al., 2017; Ren et al., 2017; Soriano and Alvaro, 2019; Li et al., 2020; Meng-jiao et al., 2020; Danakumara et al., 2021; Yang et al., 2021a) on chromosomes 5A, 5D, 6A, 6B, 6D, 7A and 7B. Previously discovered QTL for root traits and grain yield were depicted. The QTL identified in this study were labelled in red colour, and the previously studied QTL were represented by other colours (blue, yellow, green, green-cyan, magenta and blue-magenta). Blue circles represent the genomic regions of the validated QTL (*Q.rm.uwa.6AS* and *Q.rd.uwa.7BL*) positions from this study and their overlapping with other QTL from previous studies. **Table 2** is referred to the detailed QTL identified in this study on different chromosomes; GY, grain yield; SA, surface area; RL, total root length; Rdia, root diameter; RV, root volume; NNR, number of nodal roots per plant; RSA2 root surface area of fine roots (root diameter < 0.25 mm); SRL, specific root length; RM, root dry mass; and RD, rooting depth.

Mapping QTL can also identify the relationship between the traits through the co-localization of QTL (Colombo et al., 2022) which is important for plant performance improvement. *Q.rd.uwa.7BL* (*Xgwm611–Xbarc20*) for RD had a LOD of 4.00 and phenotypic variation of 13.14% (**Table 2**; **Figure 2D**) for both 500 and 1,000 permutations. *Q.rd.uwa.7BL* were co-located with previously identified QTL including *qMRL.CK-7B* (*Xbarc257.2*–*Xgwm46*) under controlled conditions. *Q.rd.uwa.7BL* also co-located with *qMRL.LP-7B* and *qMRL-7B1* (*Xbarc1181*–*Xbarc1116*) under low P (Ren et al., 2017) and well-watered conditions (Ren et al., 2012), respectively. Two other QTL under well-watered conditions—*qLR-7B* (*Wms400*–*Wms573*) (Ehdaie et al., 2016) and *Q.RL-7BL* (*AX-94528392*) (Danakumara et al., 2021) were co-located with *Q.rd.uwa.7BL*. Two drought-stress specific QTL for RD found from the RILs derived in Synthetic W7984 and Opata M85 (Ayalew et al., 2017) were co-located with *Q.rd.uwa.7BL*, suggesting that *Q.rd.uwa.7BL* may contribute to drought stress tolerance. Importantly, *Q.rd.uwa.7BL* was co-located with grain yield QTL (*Xm43p78.14*–*Xm86p65.0*) (Quarrie et al., 2005), kernel number per spike QTL, *QKNPS-DH-7B-2.1* (*Xbarc276.1*–*Xwmc396*), and thousand-grain weight QTL, *QTKW-DH-7B* (*Xgwm333*–*Xwmc10*) (Zhang et al., 2016) on chromosome 7B. These co-location evidence strongly suggest that *Q.rd.uwa.7BL* contributes to improving RD and wheat yield. However, for the first time *Q.rd.uwa.7BL* was successfully validated for RD in other populations with different genetic backgrounds. The QTL may also contribute to biotic stress tolerance: *Xgwm344*, a closely linked marker of *Q.rd.uwa.7BL* previously validated for leaf rust resistance in wheat (Zhang et al., 2020). Comparing of *Q.rd.uwa.7BL* with other previously reported co-located QTL revealed that *Q.rd.uwa.7BL* is physically located in a larger interval (329.79–700.63 Mb) than the above-mentioned QTL, except *qLR-7B* (**Figure 3**).

A number of QTL for RD have been reported on chromosome 7B (**Figure 3**) (Ren et al., 2012; Liu et al., 2013; Xu et al., 2013; Ehdaie et al., 2016; Ren et al., 2017; Ayalew et al., 2018; Li et al., 2020; Danakumara et al., 2021) under different stress conditions, suggesting that chromosome 7B harbors important genes for RD to improve stress tolerance in wheat. However, none of the QTL has been validated in other populations. In this study, *Q.rd.uwa.7BL* with a peak marker of *Xbarc50* was used to screen the validation population. Synthetic W7984 contributed to *Q.rd.uwa.7BL* for shallow RD; therefore, *Xbarc50* was validated in Synthetic W7984 × Chara hybrids (F_2_). A significant reduction in RD in the validation population confirmed the reliability of the marker performance. However, the marker could be further tested in other genotypes for application in wheat breeding. Previous studies have reported the significance of shallow RD in high yield of wheat. Under well-watered conditions, genotypes with shallow RD in durum wheat (Bellario and Jabal2), contributed to high yield. Under irrigated conditions, the shallow-rooted genotypes contributed to 20–40% higher yield than deep-rooted genotypes (El Hassouni et al., 2018). The short root length gene, *TaSRL1* (on chromosome 4A: 3.37 Mb) improved thousand grain weight as a pleiotropic effect (Zhuang et al., 2021). Importantly, it was revealed in an earlier study that despite taking up 20% less water under drought conditions, Synthetic W7984 (donor parent of *Q.rd.uwa.7BL* in our study) had higher grain numbers per spike than Opata M85 (Onyemaobi et al., 2018). Therefore, the validated marker for shallow RD from this study could be used to improve the grain yield and stress tolerance in wheat.

*Q.rm.uwa.6AS* (*Xbcd21*–*Xcmwg652*) identified in this study, was co-located with a previously identified grain yield QTL, *QGY.cgb-6A* (*Xgwm334*–*WMC297*) under both well-watered and water-stressed conditions (Liu et al., 2013) indicating its potential for drought tolerance and grain yield improvement. A meta-QTL for RM, *Root_MQTL_67* (Soriano and Alvaro, 2019), overlapped with *Q.rm.uwa.6AS* (*Xgwm334*), but was not validated. In this study, Opata M85 contributed positive alleles to *Q.rm.uwa.6AS* for increased RM. Testing the allelic performance of *Xgwm334*, in Opata M85 × Cascade hybrids (F_2_), Opata M85 had significantly higher RM than Cascade confirming the functionality of the identified QTL. Therefore, using of *Q.rm.uwa.6AS* in future MAS or other advanced genetic approaches may help improve RM, yield, and stress tolerance in wheat. The QTL could be tested in wider populations for wheat breeding. On the other hand, *Q.rm.uwa.7AL* for RM identified in this study was co-located with previously identified *QTrl.D84-7A* (*Xbarc275*) for RL under both well-watered and drought-stress conditions in a back cross population of Devon × Syn084 (Ibrahim et al., 2012). Another grain yield QTL (*Xpsp3094.1*–*Xm68p78.6*) on chromosome 7A was co-located with *Q.rm.uwa.7AL* (Quarrie et al., 2005). As no similar QTL for root traits on chromosome 6AS and 7AL were reported, *Q.rm.uwa.6AS* and *Q.rm.uwa.7AL* were novel discoveries in this study. Validation of *Q.rm.uwa.7AL* is recommended in the future.

In this study, QTL for RD (*Q.rd.uwa.5DL*) and QTL for RSA2 (*Q.rsa2.5DL*) overlapped and shared the same marker, *Xmwg900* (**Figure 2B**). These QTL also overlapped with a previously reported QTL for root volume (Ibrahim et al., 2012). Furthermore, the marker interval was found within a recently identified QTL (*IWB61072*–*IWB49479*) for grain yield (Li et al., 2018) suggesting the importance of *Q.rd.uwa.5DL* and *Q.rsa2.5DL* over other root traits and grain yield. However, there was no overlapping evidence for *Q.rl.uwa.5AL*, *Q.rd.uwa.5AL*, *Q.rdia.uwa.6AL*, *Q.rm.uwa.7AL*, or *Q.srl.uwa.6DS* discovered in this study with any previous root trait QTL (**Figure 3**) which suggesting that they are novel QTL for controlling root traits.

### Putative candidate genes on chromosomes 6A and 7B

We identified 2,323 genes in the *Q.rd.uwa.7BL* regions and 387 genes in the *Q.rm.uwa.6AS* regions. Proteins encoded by 215 genes of *Q.rd.uwa.7BL* and 34 genes of *Q.rm.uwa.6AS* were associated with the wheat root traits (**Supplementary Table S1**). In a recent review article, Halder et al. (2022) listed the number of proteins associated with the wheat root system. However, among the identified genes, proteins encoded by 21 and 13 genes in *Q.rd.uwa.7BL* (329.79–700.63 Mb) and in *Q.rm.uwa.6AS* (8.00–22.02 Mb), respectively, had roles in controlling root traits in different crops such as rice, barley, maize and soybean, and *Arabidopsis* (**Table 5**).

Phytohormones such as cytokinin play important role in root development (Aloni et al., 2006), with histidine-containing phosphotransfer (HK) and glutaredoxin proteins regulating cytokinin signaling. Transgenic plants with reduced cytokinin had greater root growth and more lateral roots than those plants with high cytokinin (Nishimura et al., 2004). HK proteins regulate phosphorylation to control the root growth of *Arabidopsis* (Hutchison and Kieber, 2007) and barley (under limited/resupply of P) (Ma et al., 2021). We found *TraesCS6A01G020400* encoded HK proteins which located very close (0.23 Mb) to the validated marker, *Xgwm334*, and *TraesCS7B01G404000* encoded glutaredoxin protein which located on the QTL for shallow RD. In *Arabidopsis*, a genotypes *AtGRXS3/4/5/8* with silenced glutaredoxin proteins had large primary roots (Patterson et al., 2016) indicating the negative role of glutaredoxin protein in RD. Glutaredoxin also play important role in stress tolerance through redox state of cell, redox dependent pathway regulation, and also improve nutrient uptake. In rice root, glutaredoxin improved arsenic (Verma et al., 2020) and salinity (Verma et al., 2021) stress tolerance. In *Arabidopsis* root, glutaredoxin improved nitrogen uptake and ammonium stress tolerance (Patterson et al., 2016).

Another protein, 3-ketoacyl-CoA synthase (KCS) condenses very-long-chain fatty acids essential for cuticular waxes and suberin production in roots (Lee et al., 2009; Kim et al., 2021). Suberin is critical role in drought and salinity stress tolerance in root (de Silva et al., 2021). For example, reduced suberin restricted root growth in *Arabidopsis* (Lee et al., 2009). A *KCS6* barley mutant with reduced cuticular waxes had reduced seminal root length but increased lateral root length (Weidenbach et al., 2015). Therefore, future exploration of the KCS encoding genes (*TraesCS6A01G024400* and *TraesCS7B01G254900*) identified in this study could be useful for improving stress tolerance in wheat through improved RM, and RD improvement. *TraesCS6A01G026500*, identified in our study, encoded lysine-specific demethylase (LSD). LSD belongs to histone demethylase (amine oxidase superfamily) which contributes to root elongation and abiotic stress tolerance through histone modification (Sun et al., 2019; Liu et al., 2022). In maize, the LSD encoding hub gene [*Zm00001d002266*: genes with top 10% correlation within a module (Liu et al., 2019)], controlled seminal root length under drought and controlled conditions (Guo et al., 2020). Transgenic *Arabidopsis* with overexpressed LSD encoding *Glyma.17G022500* improved salinity stress tolerance (Sun et al., 2019).

Few candidate genes for RD and RM encode proteins with similar functions indicating their importance for future wheat breeding. Germin-like protein (GLP) was first identified in germinating wheat grains (Bernier and Berna, 2001). However, the role of GLP genes in wheat root trait control is unclear, except for an association between GLP and cell wall modification for improved aluminum stress tolerance in wheat (Delisle et al., 2001; Houde and Diallo, 2008). GLP genes *Gs1* and *Gs2* are highly expressed in barley roots and expressed salinity stress tolerance (Hurkman et al., 1991). GLP contributed to multiple stress (e.g. as drought, heat, cold, and oxidative stress) tolerance in *Arabidopsis* and rice (Li et al., 2016). Therefore, it would be interesting to explore the genes encoding GLP for RD and RM (**Table 5**). We also found six candidate genes encoding mitochondrial transcription termination factor (mTERF) in *Q.rm.uwa.6AS*, and thaumatin-like protein encoding genes—*TraesCS6A01G021000* in *Q.rm.uwa.6AS* and *TraesCS7B01 G446200* and *TraesCS7B01G25590* in *Q.rd.uwa.7BL*. mTERF and thaumatin-like protein expressed in roots of different crops and *Arabidopsis* and play important role in abiotic and biotic stress tolerance. For example, mTERF encoding genes *shot1* and *mterf6-5* in an *Arabidopsis* mutant expressed heat tolerance (Kim et al., 2012) and salt tolerance (Robles et al., 2018), respectively. Thaumatin-like protein highly expressed in roots in barley (Iqbal et al., 2020) and *Arabidopsis*, and contributed to abiotic (e.g. drought and salt) and biotic (e.g. fungus) stress tolerance in *Arabidopsis* (Misra et al., 2016; de Jesús-Pires et al., 2020). In-silico studies of the putative candidate genes suggested that the genes expressed at different levels (0.02–360.65) in root tissues of different wheat cultivars. Important genes, *TraesCS7B01G404000*, *TraesCS7B01G254900* and *TraesCS7B01G446200*, from *Q.rd.uwa.7BL* showed high (124.32) to low (0.89) gene expression while important genes from *Q.rm.uwa.6AS*, *TraesCS6A01G024400*, *TraesCS6A01G021000* and *TraesCS6A01G020400* showed low (0.20–0.11) gene expression in roots of previously studied wheat cultivars (**Supplementary Table S2**). However, the study conditions and the studied root traits of the previous studies may cause variation in gene expression, which could be confirmed by future gene expression approaches. Moreover, after further functional validation, the identified putative candidate genes may be useful for wheat breeding programs for root improvement.

## Conclusion

Eleven QTL were identified on chromosomes 5A, 5D, 6A, 6B, 6D, 7A and 7B for seven root traits in bread wheat suggesting that wheat chromosome groups 5, 6 and 7 harbor major QTL/genes for root traits. *Q.rd.uwa.7BL* co-located with previously identified grain yield and biotic and abiotic stress tolerance markers. *Q.rm.uwa.6AS*, is a novel QTL for RM. Validation studies confirmed the functionality of *Q.rd.uwa.7BL* and *Q.rm.uwa.6AS* in two independent F_2_ populations. The putative candidate genes located in the validated QTL encode important proteins for root traits in other crops. Further gene validation is required to confirm their role in wheat breeding. The identified and validated QTL/markers and putative candidate genes in this study provide a genetic foundation for marker-assisted breeding of root traits in bread wheat.

## Data availability statement

The original contributions presented in the study are included in the article/**Supplementary Material**. Further inquiries can be directed to the corresponding authors.

## Author contributions

Investigation, formal analysis, and preparation of the original draft: TH. Supervision: KS, GY, HL, and YC. Writing—review and editing: KS, GY, HL, and YC. All authors have read and agreed to the published version of the manuscript. All authors contributed to the article and approved the submitted version.
